# Nutritional Ketosis as a Therapeutic Approach in Critical Illness: A Systematic Review

**DOI:** 10.7759/cureus.65455

**Published:** 2024-07-26

**Authors:** Rana A Abdelrahim, Sai Rohit R Mekala, Krishna V Polepalli, Vemparala Priyatha, Chimezirim Ezeano, Esraa M AlEdani, Sondos T Nassar

**Affiliations:** 1 Medicine, The Royal College of Surgeons in Ireland-Bahrain, Busaiteen, BHR; 2 Internal Medicine, California Institute of Behavioral Neurosciences and Psychology, Fairfield, USA; 3 Gastroenterology and Hepatology, Mayo Clinic, Rochester, USA; 4 School of Medicine, Armed forces Medical College, Pune, IND; 5 Medicine, California Institute of Behavioral Neurosciences and Psychology, Fairfield, USA; 6 Internal Medicine, All India Institute of Medical Sciences, Bhubaneswar, Bhubaneswar, IND; 7 Department of Pediatrics and Women's Health, University of North Texas Health Science Center, Fort Worth, USA; 8 Dermatology, California Institute of Behavioral Neurosciences and Psychology, Fairfield, USA; 9 Medicine and Surgery, Jordan University of Science and Technology, Amman, JOR

**Keywords:** nutrition, icu, ketone bodies, high-fat very low-carbohydrate diet, ketosis, therapeutic ketosis, ketogenic diet, nutritional ketosis, intensive care, critical illness

## Abstract

Critical illness encompasses the dysfunction of vital organs, the risk of death, and potential reversibility; it is a major cause of morbidity and mortality globally. The pathophysiology underlying many critical illnesses includes bioenergetic failure, inflammation, and oxidative stress. This systematic review aims to explore the use of nutritional ketosis (ketogenic feeds and exogenous ketone body administration) as a potential therapy, affecting the aforementioned pathways in patients with critical illnesses. This study was conducted, following the Preferred Reporting Items for Systematic Reviews and Meta-Analyses (PRISMA) 2020 guidelines. The search was conducted, systematically using PubMed, PubMed Central (PMC), Google Scholar, and the ScienceDirect databases in February 2024. The inclusion criteria were set to free full-text articles published within the past five years: human and animal studies, literature reviews, systematic reviews, meta-analyses, observational studies, randomized controlled trials, case reports, book chapters, gray literature, studies investigating adult samples, and articles in the English language. Exclusion criteria included pediatric studies as the topic has been studied more extensively in children, and this review aims to explore potential benefits in adult patients. The search was conducted through four databases; after the screening process, the remaining studies were assessed through quality appraisal tools appropriate to each study type. In the end, 11 studies were selected, including eight narrative reviews, one cohort study, one animal study, and one randomized controlled trial. The results of this review demonstrated that nutritional ketosis has potential safe and effective benefits for humans and animals. Nutritional ketosis shows therapeutic benefits for a vast variety of complications commonly associated with critical illness, status epilepticus, sepsis, viral infections, and glycemic control. In the end, both randomized and nonrandomized clinical trials are necessary for more conclusive findings.

## Introduction and background

The annual global incidence of critical illness is estimated to be around 30-45 million [1], with the mortality rate of patients admitted to the Intensive Care Unit (ICU) estimated to be around 40% [2]. Critical illness can be defined as the dysfunction of vital organs, a high risk of impending mortality without intervention, and the possibility of the critical state being reversed [1]. The aim of nutrition in this population is to decrease the detrimental consequences of the illness and improve patient outcomes; however, the optimal nutrition strategy remains undetermined [3]. Newer approaches, such as ketogenic diets (KD), have gained interest over the past few decades. 

**Figure 1:**
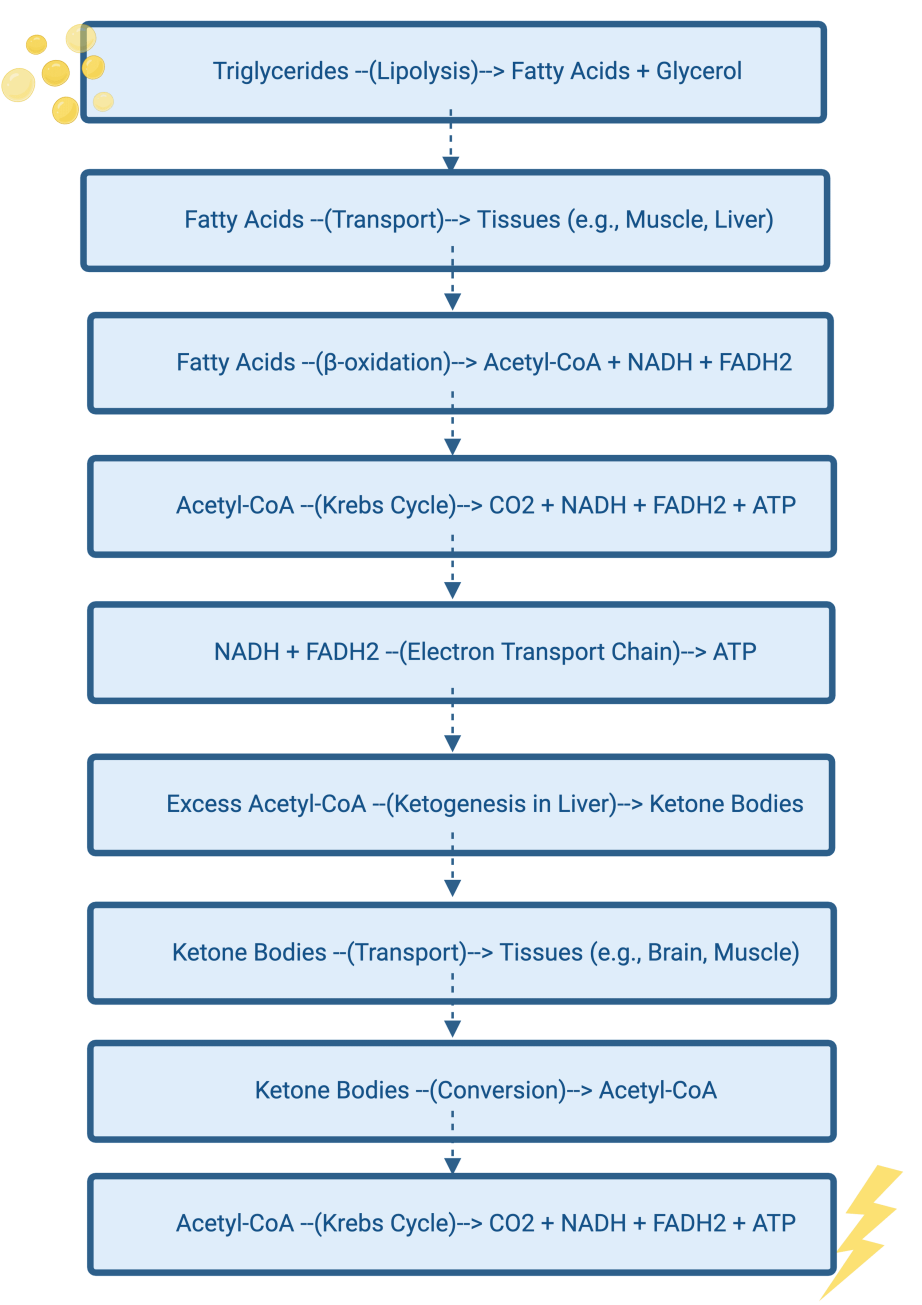
Lipid conversion to ketone bodies and energy Figure created through BioRender.com. β-Oxidation: Beta-oxidation; Acetyl-CoA: Acetyl coenzyme A; NADH: Nicotinamide Adenine dinucleotide (reduced form); FADH2: Flavin adenine dinucleotide (reduced form); ATP: Adenosine triphosphate; CO2: Carbon dioxide.

The ketogenic diet is defined as a high-fat, very low-carbohydrate diet, emulating the metabolic effects of fasting and producing ketone bodies such as β-hydroxybutyrate (BHB) and acetoacetate (AcAc) without substantial caloric deprivation [4]. Normally, during fasting or caloric deprivation, the expenditure of blood glucose reduces insulin levels, stimulating ketogenesis [5]. During ketogenesis, triglycerides are broken down into fatty acids that are converted into ketone bodies in the liver [5]. These ketone bodies are then converted into acetyl-CoA and adenosine triphosphate (ATP) and utilized as energy in active tissues like the brain and muscle [5]. In summary, triglyceride breakdown can form ketone bodies that provide ATP as an alternative energy source during caloric deprivation [5]. A simplified description of this process is included in Figure 1 [5].

Likewise, with the administration of a ketogenic diet, the low-carbohydrate and high-fat content cause a similar response; emptying the body’s glucose reserve stimulates ketogenesis, producing ketone bodies that act as an alternative source of energy [6]. Under basal conditions, ketone body serum concentrations are around 0.1 to 0.4 mM [7]. After short-term fasting of 2 to 3 days, ketone body concentrations range from 1 to 4 mM [7]. After prolonged fasting of 17 to 24 days, levels range between 7 and 9 mM [7]. With KDs, ketone body levels in the serum can increase to higher than 5 mM [7]. The rationale behind the benefits of nutritional ketosis (through a ketogenic feeding diet or exogenous ketone body administration) and the improvement of critical illnesses is multifaceted. Ketones inhibit inflammasomes, preventing the secretion of pro-inflammatory cytokines, hence exhibiting beneficial anti-inflammatory effects [5]. Additionally, ketones control mitophagy and increase mitochondrial biogenesis, providing beneficial antioxidant effects and preserving cellular energy [5]. Moreover, nutritional ketosis may improve metabolic stability by reducing insulin resistance and hyperglycemia [8]. Inflammation, oxidative damage, and metabolic instability are overarching issues in critical illness, and therefore, a diet mitigating these effects is of great interest. Current literature conveys nutritional ketosis to be beneficial in various conditions, including sepsis, brain injury, refractory status epilepticus, glycemic control, and respiratory infections [9-13]. Due to these anti-inflammatory, anti-oxidant, and metabolic effects, recent research advancements have also been exploring the effects of ketosis in malignancy and neurodegenerative diseases [14,15]. 

Nutritional ketosis seems to provide promising benefits in animal studies, pediatric studies, and smaller clinical studies of the adult population, but there is a lack of substantial data to recommend the use of ketogenic diets in adults with critical illnesses. In this systematic review, we explored the use of nutritional ketosis as a therapeutic approach in critically ill adults and its effects on patient outcomes.

## Review

Methods

This study was conducted following the Preferred Reporting Items for Systematic Reviews and Meta-Analyses (PRISMA) 2020 guidelines [16].

Eligibility Criteria

The question for this systematic review was generated based on the participants, intervention, and outcome (PIO) elements: participants were adult patients with a critical illness or admitted to the ICU; the intervention was nutritional ketosis; and the outcome was any improvement of critical illness outcomes. Furthermore, inclusion and exclusion criteria were applied. Inclusion criteria: free full-text articles published within the past five years (2019-2024), human and animal studies, adult study populations, articles in the English language, literature reviews, systematic reviews, meta-analyses, observational studies, randomized controlled trials (RCTs), case reports, book chapters, and gray literature. Exclusion criteria: pediatric studies and papers published in languages other than English. 

Databases and Search Strategy

The search was conducted systematically using PubMed, PubMed Central (PMC), Google Scholar, and ScienceDirect databases. The last search of all databases was performed in February 2024. The key terms used in the search engines were Nutritional Ketosis, Ketogenic Diet, Ketogenic Nutrition, Ketogenic Feed, Ketone-based Nutrition, Low-carbohydrate Diet, Carbohydrate-reduced Nutrition, High-fat, Low-carbohydrate Diet, Ketogenic Therapy, Ketogenic Intervention, Ketogenic State, Ketogenic Treatment, Critically Ill Patients, Critical Illness, Critical Condition, Intensive Care Unit, Critical Care Unit, Intensive Care, Critical Care, Critical State. The Medical Subject Heading (MeSH) strategy was employed in PubMed. Table 1 provides details of the search strategy used in each database.

**Table 1:** Description of search strategy and results by database

Databases	Keywords	Search Strategy	Filters	Search result
PubMed	Nutritional Ketosis, Ketogenic Diet, Ketogenic Nutrition, Ketogenic Feed, Ketone-based Nutrition, Low-carbohydrate Diet, Carbohydrate-reduced Nutrition, High-fat, Low-carbohydrate Diet, Ketogenic Therapy, Ketogenic Intervention, Ketogenic State, Ketogenic Treatment, Critically Ill Patients, Critical Illness, Critical Condition, Intensive Care Unit, Critical Care Unit, Intensive Care, Critical Care, Critical State	Nutritional Ketosis OR Ketogenic Diet OR Ketogenic Nutrition OR Ketogenic Feed OR Ketone-based Nutrition OR Low-carbohydrate Diet OR Carbohydrate-reduced Nutrition OR High-fat, Low-carbohydrate Diet OR Ketogenic Therapy OR Ketogenic Intervention OR Ketogenic State OR Ketogenic Treatment OR ( "Diet, Ketogenic/adverse effects"[Mesh] OR "Diet, Ketogenic/classification"[Mesh] OR "Diet, Ketogenic/instrumentation"[Mesh] OR "Diet, Ketogenic/methods"[Mesh] OR "Diet, Ketogenic/mortality"[Mesh] OR "Diet, Ketogenic/standards"[Mesh] OR "Diet, Ketogenic/statistics and numerical data"[Mesh] OR "Diet, Ketogenic/trends"[Mesh] ) AND Critically Ill Patients OR Critical Illness OR Critical Condition OR Intensive Care Unit OR Critical Care Unit OR Intensive Care OR Critical Care OR Critical State OR ( "Critical Illness/classification"[Mesh] OR "Critical Illness/epidemiology"[Mesh] OR "Critical Illness/mortality"[Mesh] OR "Critical Illness/rehabilitation"[Mesh] OR "Critical Illness/therapy"[Mesh] ) - 1,082,137	Custom filter: 1.01.2019 - 01.01.2024 + Free Full text + Adults 19+ years + Humans and Animals + All article types selected. Only results until 01/01/2024 will be included.	6,814
PMC	Nutritional Ketosis, Ketogenic Diet, Critical Illness	((Nutritional Ketosis) OR Ketogenic Diet) AND Critical Illness - 1,909	Five years (2019/01/01 - 2024/01/01).	1,358
Google Scholar	Nutritional Ketosis, Ketogenic Diet, Ketogenic Nutrition, Ketogenic Feed, Ketone-based Nutrition, Low-carbohydrate Diet, Carbohydrate-reduced Nutrition, High-fat, Low-carbohydrate Diet, Ketogenic Therapy, Ketogenic Intervention, Ketogenic State, Ketogenic Treatment, Critically Ill Patients, Critical Illness, Critical Condition, Intensive Care Unit, Critical Care Unit, Intensive Care, Critical Care, Critical State	"Nutritional Ketosis" OR "Ketogenic Diet" OR "Ketogenic Nutrition" OR "Ketogenic Feed" OR "Ketone-based Nutrition" OR "Low-carbohydrate Diet" OR "Carbohydrate-reduced Nutrition" OR "High-fat, Low-carbohydrate Diet" OR "Ketogenic Therapy" OR "Ketogenic Intervention" OR "Ketogenic State" OR "Ketogenic Treatment" AND "Critically Ill Patients" OR "Critical Illness" OR "Critical Condition" OR "Intensive Care Unit" OR "Critical Care Unit" OR "Intensive Care" OR "Critical Care" OR "Critical State" - 2,810	Five year cutoff 2019-2024. Only results until 01/01/2024 will be included.	1,350
ScienceDirect	Nutritional ketosis, Ketogenic Diet, Critically Ill Patients, Critical Illness	"Nutritional ketosis" OR "Ketogenic Diet" AND "Critically Ill Patients" OR "Critical Illness" - 27,781	Five year cut off (2019-2024) + English + Open Access and Open Archive + Review Articles+ Research Articles+ Book Chapters + Case Reports. Only results until 01/01/2024 will be included.	2,523

All references were collected and grouped with EndNote. The removal of duplicates was completed both by EndNote and manually. Subsequently, the remaining articles were screened by titles and abstracts, excluding any records irrelevant to the question. Full-text articles were obtained. To reduce the risk of bias in this study, articles that were successfully obtained were evaluated for quality using the appropriate tools for quality appraisal.

Risk of Bias in Individual Studies

The nominated articles were assessed for risk of bias, ensuring quality appraisal through tools depending on each study type: narrative reviews: Scale for the Assessment of Narrative Review Articles 2 (SANRA 2); cohort studies: Newcastle Ottawa Scale (NOS); animal studies: Systematic Review Centre for Laboratory Animal Experimentation's (SYRCLE) risk of bias tool; and randomized control trials: Cochrane Risk of Bias 2 List [17-20]. For all assessment tools, a passing score of 70% was required for articles to pass the quality appraisal.

Data Collection Process

Data on the improvement of critical illness, any additional symptoms, and changes in morbidity and mortality were thoroughly examined in the finalized articles. As the topic lacks a large number of human clinical trials and studies, animal studies were included in the search. Co-authors contributed to the editing of the paper.

Results

Study Selection and Quality Assessment

**Figure 2:**
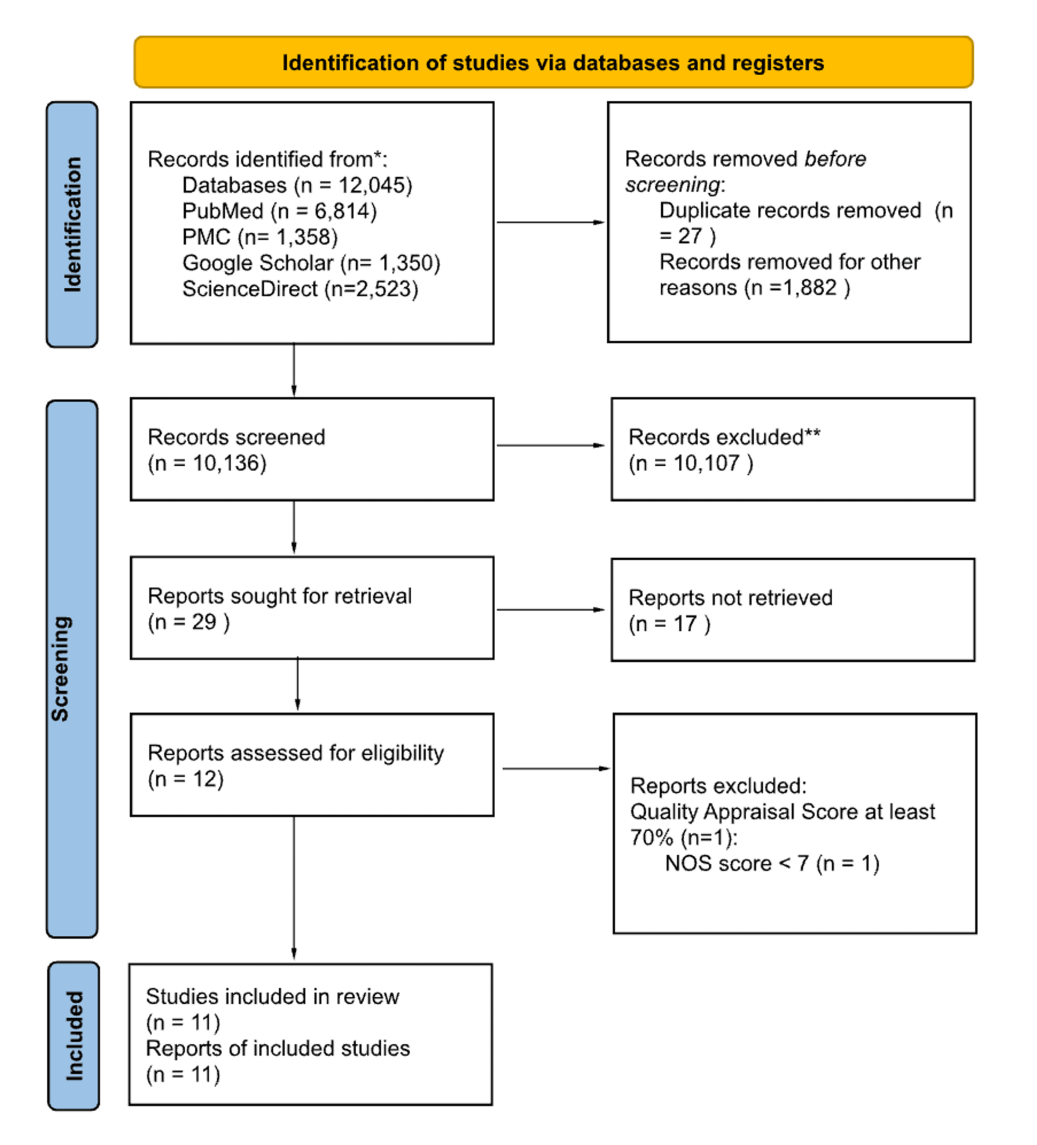
Screening and study selection PRISMA flow diagram The PRISMA 2020 statement [16]. PRISMA: Preferred Reporting Items for Systematic Reviews and Meta-Analyses; PMC: PubMed Central; NOS: Newcastle Ottawa Scale.

The start of the database search yielded 12,045 potential papers. Following the removal of duplicates and nonexistent results, 10,136 titles were retained. Next, during the screening by titles, abstracts, eligibility criteria, and relevance to the PIO elements of this study, 10,107 reports were excluded. The remaining 29 reports were sought for retrieval. Twelve reports were successfully retrieved and assessed for quality by the first author. Finally, 11 reports scored greater than 70% and were included in this study. The 11 reports include eight narrative reviews, one cohort study, one animal study, and one randomized controlled trial. Figure 2 presents a flow diagram detailing the screening and study selection process.

Tables 2-5 present how each report was assessed according to its respective assessment tool, and the final result. Table 2 details the assessment of narrative reviews through the SANRA 2 checklist [17]. All eight narrative reviews passed the quality appraisal. Table 3 summarizes the use of the NOS Assessment Tool in evaluating the two observational cohort studies [18]. Only one study scored higher than 70% and was included in this review. Table 4 demonstrates the quality assessment of one animal study based on the SYRCLE assessment tool [19]. The study scored 7/10 based on the criteria and was included in this review. Lastly, Table 5 details the quality assessment using the Cochrane RoB2 (Risk of Bias 2) assessment tool for the randomized controlled trial included in this review [20]. Due to all criteria being low-risk, the final risk of bias was deemed low, and this report was included in our review.

**Table 2:** Results of the SANRA 2 assessment tool for narrative reviews by review authors Passing score: 9/12. SANRA 2: Scale for the Assessment of Narrative Review Articles 2 [17].

Author, date	Justification of the article’s importance for the readership	Statement of aims or formulation of the question	Description of the literature search	Referencing	Scientific reasoning	Appropriate presentation of data	Sum	Pass/Fail
Cai et al., 2022 [11]	2	2	1	2	2	2	11	Pass
Burslem et al., 2022 [12]	2	2	2	2	2	2	12	Pass
Stubbs et al., 2020 [13]	2	2	0	2	2	2	10	Pass
Husari et al., 2020 [21]	2	2	1	2	1	2	10	Pass
Muniz-Santos et al., 2023 [22]	2	2	0	2	2	2	10	Pass
Vandewalle and Libert, 2022 [23]	2	2	0	2	2	1	9	Pass
Wischmeyer et al., 2023 [24]	2	2	2	2	2	2	12	Pass
Katz et al., 2021 [25]	2	2	0	2	2	2	10	Pass

**Table 3:** Results of the NOS assessment tool for observational studies by review authors Studies assessed are retrospective cohort studies. Passing score: 7/9. Y: yes; N: no; N/A: not applicable; NOS: Newcastle Ottawa Scale [18].

Author, date	Representativeness of exposed cohort	Selection of nonexposed cohort	Ascertainment of exposure	Demonstration of absence of outcome at the start	Comparability of cohorts (2 points)	Assessment of outcome	Follow-up duration	Adequacy of follow-up	Pass/Fail
Kaul et al., 2022, [26]	Y: Truly representative of patients.	N: No, all patients were exposed.	Y: Secure records.	N/A*	N: No, all patients were exposed.	Y: Record linkage.	Y: Yes, long enough.	N: Not adequate.	Fail
Koh et al., 2022 [27]	Y: Truly representative of patients.	Y: Drawn from the same community.	Y: Secure records.	N/A*	Y: The study controls for non-ketogenic diet patients.	Y: Record linkage.	Y: Yes, long enough.	N: Not adequate, missing data at 3 months accounted for 30.7%.	Pass

**Table 4:** Results of the SYRCLE assessment tool for animal studies by review authors Passing score: 70%. * = point subtracted; N/A: not applicable; SYRCLE: Systematic Review Centre for Laboratory animal Experimentation [19].

Author, year	Sequence generation	Baseline characteristics	Allocation concealment	Random housing	Blinding	Random outcome assessment	Blinding	Incomplete outcome data	Selective outcome reporting	Other sources of bias	Include/exclude
Weckx et al., 2022 [28]	Yes	Yes	Yes	N/A (not mentioned)*	No*	Yes	No*	No	No	No	Include

**Table 5:** Results of the Cochrane RoB2 assessment tool for randomized controlled trials by review authors RoB: risk of bias; LR: low risk; MR: moderate risk; HR: high risk [20].

Authors and year of publication	Random Allocation	Intervention Non-adherence	Incomplete Results	Inadequate Assessment of Outcomes	Selective Reporting	Final RoB Judgement	Result
Mcnelly et al., 2023 [29]	LR	LR	LR	LR	LR	LR	Include

Discussion

The Rationale and Safety of Therapeutic Ketosis in Critical Illness

As this systematic review aimed to investigate critically ill subjects, limited evidence was expected as this is a novel therapeutic approach for higher-risk patients. As such, we defined nutritional ketosis to include ketogenic feeding formulas, exogenous ketone body administration, and low-carbohydrate, high-fat diets. As such, the formulations differ between studies. The classic ketogenic enteral nutrition formula most commonly uses a 4:1 or 3:1 ratio of fat (long-chain saturated triglycerides, delivering 90% of the energy) to carbohydrates and protein [11]. As for ketogenic parenteral or intravenous nutrition, preparations are largely based on pediatric studies [11]. Based on pediatric practice guidelines on ketogenic parenteral nutrition, a maximum of 4 g/kg of fat is recommended daily and a reduced fat/non-fat ratio of merely 4:1 in comparison to enteral nutrition [11]. The fat components in parenteral preparations consist of medium-chain triglyceride emulsion (20% fat emulsion) and long-chain triglyceride emulsion (20% fat emulsion) with an amino-acid intravenous nutrient solution (6.7% or 10% concentration) in a 1:3 ratio [11]. The maximum daily maintenance intake of carbohydrates may be determined based on prior intestinal intake if the patient previously received enteral nutrition [11]. Lastly, the administration of bio-identical exogenous ketones depends on their availability [13]. One type of exogenous ketone compound is ketone salts (which cause around a 1 mM BHB increase) [13]. Medium-chain triglycerides induce a longer-lasting ketosis (approximately 0.5-1 mM BHB increase) without the added salt [13]. Ketone esters, containing ketones and their precursors, increase blood BHB levels more significantly (around 1-5 mM BHB increase) [13]. Moreover, as human studies are limited, our study reviewed animal studies to provide further evidence and clearer pathophysiological data. To understand how a ketogenic diet may be useful in critical illness, it is imperative to primarily delve into the overarching physiological implications that are commonly shared by critical illnesses. 

Critical illness can be defined as an illness that includes the dysfunction of vital organs, a high risk of impending mortality in the absence of care, and the possibility of reversal [29]. Critical illness results in bioenergetic failure through disrupted cellular metabolism and the inadequate consumption of glucose, fatty acids, and amino acids [29]. Normally, glucose-derived pyruvate produces acetyl-CoA, a crucial molecule for energy generation in the tricarboxylic acid cycle (TCA, or Krebs cycle) [29]. However, tissue inflammation and hypoxia lead to what is known as the Pasteur effect [29]. Precisely, this effect is seen during periods of illness, where the body prioritizes the generation of lactate instead of acetyl-coA from substrates arising from glucose [29]. The second characteristic of critical illness is the reduction of fatty acid oxidation in peripheral mitochondria [29]. Third, critical illness impairs the use of amino acids for pyruvate reconstitution (through the Cahill cycle) during starvation [29]. With critical illness impairing the effective utilization of glucose, fatty acids, and amino acids as energy substrates, a detrimental state of bioenergetic failure ensues [29]. 

In periods of bioenergetic failure and physiological stress, fatty acids can be metabolized by the liver into ketone bodies, including BHB and AcAc, which are then used as substrates for adenosine triphosphate (ATP) production [29]. The breakdown of ketone bodies into acetyl-CoA, a crucial energy substrate, takes place in extra-hepatic mitochondria [29]. The rate-limiting step of this reaction is defined by the activity of the enzyme succinyl-CoA-oxoacid transferase [29]. Importantly, unlike pyruvate dehydrogenase kinase, this enzyme is not affected by inflammation or hypoxia [29].

**Figure 3:**
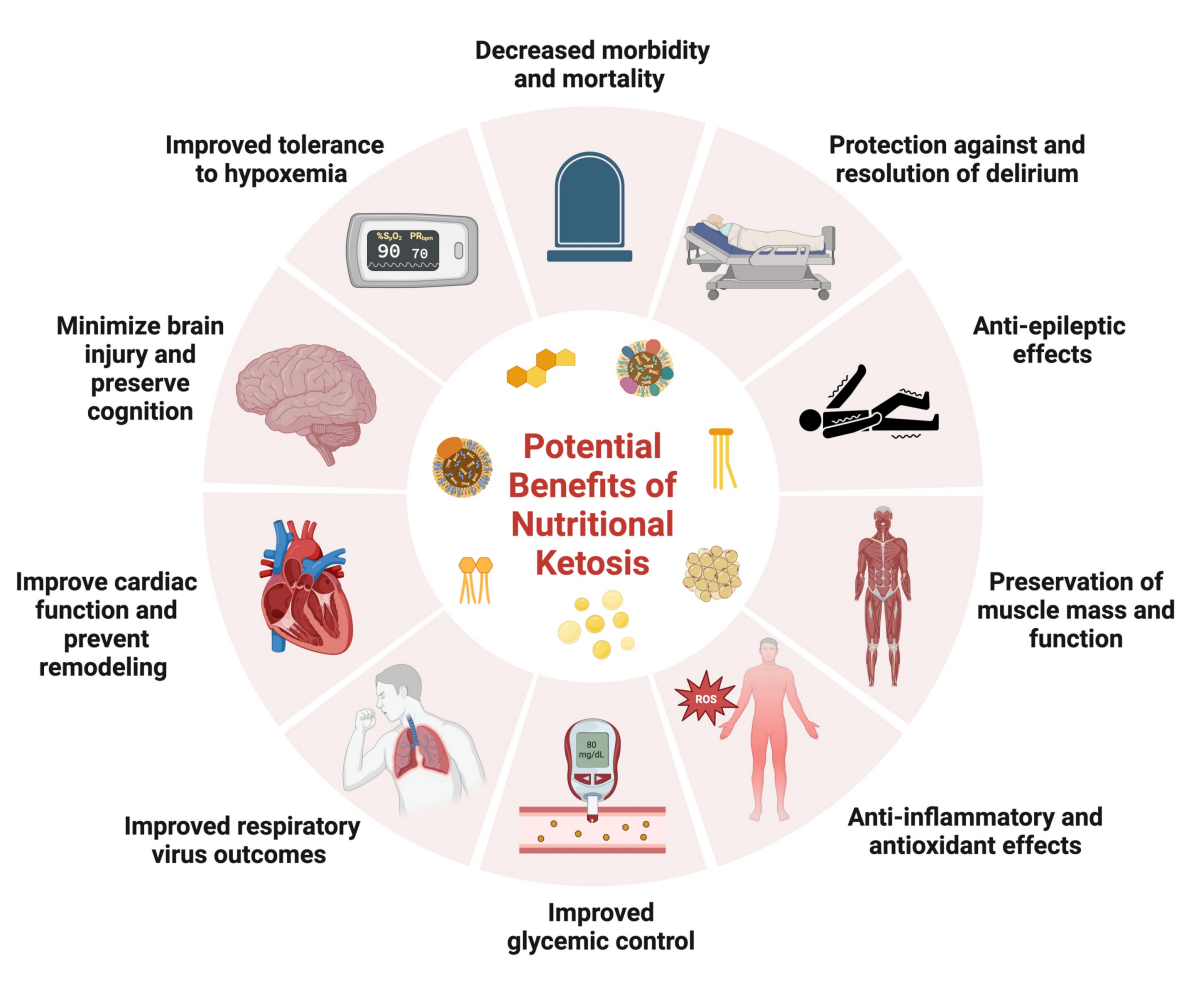
Overview of potential benefits of nutritional ketosis in critical illness Figure created through BioRender.com. ROS: reactive oxygen species.

Therefore, ketone bodies can be a useful energy resource for tissue inflammation and hypoxia. Additionally, when the body is starved, the brain depends on ketone bodies for energy [29]. Ketone bodies can supply about 50% of the entire body’s basal energy requirements. Hence, the brain’s substantial energy demands can be met while preserving muscle [29]. Another vital organ that can use ketone bodies for energy is the heart. In diabetic patients, ketone bodies are utilized for the synthesis of ATP in cardiac tissue [29]. In the lungs, the ketone body, BHB, reprograms T-cells to enhance their immunologic function in patients with acute respiratory distress syndrome (ARDS) [29]. Therefore, a ketogenic diet may not only help provide alternative energy in critical illnesses but may also aid in attenuating the detrimental complications of the illness [29]. Moreover, critical illness causes the loss of 2-3% of patients’ muscle mass each day, causing prolonged hospital stays, long-term functional disability, and death [29]. This complication has been refractory to high-protein, high-energy nutrition and all types of rehabilitation [29]. Muscle wasting is a consequence of decreased ATP production, and the use of a ketogenic diet may reduce the loss of muscle mass [29]. Evidently, nutritional ketosis presents pleiotropic benefits. Figure 3 provides an overview of some of the potential benefits of nutritional ketosis relevant to critical illness, to be discussed in detail in this review.

While nutritional ketosis seems promising, its application in clinical practice must be safe and feasible. The timing of the initiation of nutrition and feeding is a topic of great interest in critical care medicine. Evidence from a multitude of intensive care unit trials presents diets that alternate feeding and fasting intervals as potentially better than continuous delivery [24]. The rationale behind the protective benefits of intermittent feeding entails the generation of a fasting response, with increased autophagy and ketogenesis promoting cellular recovery [24]. During heavy exercise, ketogenic diets enhance ATP production, reduce the breakdown of muscle, and boost physical performance [29]. Having established that a ketogenic state serves a protective and beneficial role by providing an alternative energy source, we ask: can the administration of a ketogenic diet be used as a therapy in critical illness? 

A randomized control feasibility trial was conducted to confirm the safety of administering ketogenic enteral feeds to critically ill, mechanically ventilated patients with multi-organ failure [29]. The results showed enhanced blood sugar control in patients receiving ketogenic feeding compared to those on standard enteral feeding [29]. Patients in the ketogenic diet (KD) group experienced fewer hypoglycemic episodes (0.0% vs. 1.6%) and also needed fewer exogenous international units of insulin 0 (interquartile range 0-16) vs. 78 (interquartile range 0-412) [29]. However, the KD group had a slightly larger number of diarrhea episodes per day (53.5% vs. 42.9%) [29]. The study concluded that sustained ketosis through ketogenic enteral feeding is safe, achievable, and tolerated well by patients with critical illness and multi-organ failure [29]. Moreover, the benefits of this metabolic profile on critical illness outcomes necessitate future prospective trials [29]. Table 6 below provides a brief overview of the studies, study samples, modality of nutritional ketosis, aim, and results of all papers to be discussed further in this systematic review.

**Table 6:** Studies of nutritional ketosis in critical illness and results SE: status epilepticus; LCHF: low‐carbohydrate, high‐fat.

Study Reference	Study Sample	Nutritional Ketosis Technique	Aim	Results
Cai et al., 2022 [11]	Adult status epilepticus (SE) patients	Ketogenic diet (enteral, parenteral/intravenous)	Explore the ketogenic diet as an anti-epileptic for SE adult patients	↑ benefits in refractory SE, super-refractory SE, drug-resistant SE ↓adverse reactions compared to standard treatment.
Burslem et al., 2022 [12]	Adult humans with critical illness	Low‐carbohydrate, high‐fat enteral formula (LCHF)	Explore glycemic control using LCHF formulas vs. standard formulas in critical illness	↑ glycemic control in critically ill, diabetic, and hyperglycemic patients.
Stubbs et al., 2020 [13]	Humans and animals	Ketone bodies	Explore potential ketone body therapy for severe respiratory viral infections	↑ Benefits in viral respiratory infections ↑ Protection of vital organs and muscle ↓ Inflammation, hypoxic damage, glycemic instability.
Husari et al., 2020 [21]	Adult patients with epilepsy	Various ketogenic diets	Explore the ketogenic diet as an anti-epileptic for adult patients	↑ benefits in super-refractory SE and other epilepsy subsets.
Muniz-Santos et al., 2023 [22]	Humans and animals with sepsis	Not applicable, pathophysiological mechanisms explored	Explore the relationship between lipid oxidation and sepsis inflammation and metabolic instability	↓ Fatty acid oxidation in sepsis + ↑ Fatty acid blood levels.
Vandewalle and Libert, 2022 [23]	Humans and animals	Pathophysiological mechanisms and ketone bodies explored	Explore pathways and potential therapeutic targets in sepsis	Ketone bodies ↓ inflammation.
Wischmeyer et al., 2023 [24]	Humans with critical illness	Fasting intervals	Explore recommendations for personalized nutrition in critical care	↑ Cellular recovery through fasting-induced autophagy and ketogenesis ↑ Potential muscle anabolism ↓ Circadian rhythm disturbance. Personalized duration of fasting needed.
Katz et al., 2021 [25]	Adults with SE	Ketogenic Diet	Explore the benefits and challenges of ketogenic diet in SE in critical care	Ketogenic diet is safe and effective but recommendations during initiation and maintenance should be considered.
Koh et al., 2022 [27]	Adults with refractory SE	Ketogenic Diet	Explore the role of the ketogenic diet in seizure cessation and functional disability	↑ Neuroprotection in severe refractory SE with few remaining therapies ↓ Disability in higher-risk patients.
Weckx et al., 2022 [28]	Mouse model with sepsis	Exogenous ketone esters	Explore effects of ketone esters on sepsis-induced muscle force changes	↓ Sepsis-induced muscle weakness ↓ Adverse effects and toxicity with ester infusion vs. salts.
Mcnelly et al., 2023 [29]	Adults with multi-organ failure on mechanical ventilation in the intensive care unit	Ketogenic enteral feeding	Explore the effects, feasibility, and safety of ketogenic feeding	Ketogenic feeding was well-tolerated and safe and a distinctive metabolic profile was achieved with feeding.

Therapeutic Ketosis in Status Epilepticus

Since the mid-1990s, the KD has been accepted as an effective treatment for epilepsy in children [11]. A substantial number of pediatric guidelines exist, and KDs have proven to be a useful and safe treatment for epilepsy in general [11]. However, evidence to support recommendations for adults in more critical states, such as drug-resistant epilepsy (DRE), status epilepticus (SE), refractory status epilepticus (RSE), and super refractory status epilepticus (SRSE), remains scarce [11]. Status epilepticus (SE) is a neurologic emergency that leads to death 20% to 57% of the time [11]. It is defined as seizures continuing for longer than five minutes or two or more seizures with failure of consciousness recovery between each seizure [11]. Severe forms of SE include super refractory status epilepticus (SRSE) when seizures last ≥24 hours with anesthetic or recur with anesthetic weaning, and refractory status epilepticus (RSE) when seizures remain even after administration of a first- and second-line medication [11]. 

A recently published narrative review highlights the current progress and clinical application of KD in adults [11]. Specifically, data from various case reports and series highlights that the use of KDs as early therapy for drug-resistant epilepsy (DRE) yields an improvement (≥ 50% decrease from baseline) at a rate between 21% and 86% [11]. Moreover, referencing data from 13 case reports and series, 82.3% reached SRSE and RSE cessation following 4-25 days of a KD [11]. According to a recent systematic review, 81.6% of SE, SRSE, and RSE patients (31/38 patients) in four different observational studies effectively resolved seizures through the use of KD [11]. The narrative review concludes that KD has desirable benefits for adult SE patients. In comparison to anesthetic sedatives and traditional anti-seizure drugs, KD is safer and leads to fewer side effects [11]. 

Having established the potential efficacy of KD in SE, an exploratory retrospective cohort study using moderation analysis was conducted to accurately identify the characteristics of adult patients most likely to benefit from this intervention [27]. The study consisted of 140 patients with RSE, of whom 32 received a KD [27]. Of patients receiving KD, 81% reached seizure resolution, and the modified Rankin scale (mRS) score (a measurement of the degree of disability) was used to evaluate differences in the KD’s efficacy based on various clinical characteristics [27]. The retrospective study concluded that KD contributed to a decrease in the mRS score at discharge in older patients, patients with greater scores of seizure severity, patients taking continuous intravenous anesthetic therapy (CIVAD), and patients with SRSE [27]. At three months, only seizure severity scores and older age were correlated with a KD-moderated mRS score [27]. Overall, the study postulated KD’s neuroprotective effect to be beneficial in RSE patients of the greatest severity, with fewer therapeutic options available [27]. 

Furthermore, a recent narrative review on KD therapies for adults highlights additional evidence of therapeutic KD application in a variety of status epilepticus subsets [21]. The study suggests that the early start of a KD during RSE may prevent the development of SRSE [21]. Further evidence from the case series presents the beneficial use of KD in New-Onset Refractory Status Epilepticus (NORSE) and Febrile Infection-Related Epilepsy Syndrome (FIRES) as a therapeutic option [21]. A few retrospective studies were reviewed, including a study from Johns Hopkins, which presented that 70% of NORSE patients achieved cessation of status epilepticus with a KD [21]. The positive therapeutic effects of KD in NORSE and FIRES are accompanied by a reduction of proinflammatory cytokines and chemokines (namely, TNF-α, IL-1, and IL-6 and CD4 cell reduction), enhanced mitochondrial activity, and increased antioxidant activity [21]. 

Specifically, KD’s effects on brain energy metabolism, oxidative stress, neurotransmitters, and ion channels are postulated to mediate its efficacy as an antiepileptic treatment [11]. As discussed previously, ketone bodies can cross the blood-brain barrier and serve as an energy source for the brain [11]. Additionally, the neurotransmitter γ-aminobutyric acid (GABA) inhibits the start and progression of seizure activity [11]. The KD stimulates the production and decreases the breakdown of GABA, and a similar effect is seen with a higher concentration of agmatine (another inhibitory neurotransmitter) [11]. KD also modulates the sensitivity of ion channels, multiplying the seizure threshold [11]. Furthermore, KD’s generation of polyunsaturated fatty acids stimulates peroxisome proliferator-activated receptors (causing antioxidant effects and anti-inflammatory responses), builds energy reserves, modulates mitochondrial genes, and stabilizes activity at the synapses, diminishing the excessive excitement seen in epilepsy [11]. Of note, these critical patients have a higher likelihood of multi-organ failure and infection due to continued seizures, airway support, prolonged sedation, and concurrent use of various anti-seizure medications [11]. Here, KD can help with the earlier cessation of sedatives, allowing spontaneous breathing and ameliorating the side effects of medications and their effects on circulation, further enhancing clinical outcomes [11]. 

There are a few important considerations for implementing a KD for SE in the intensive care setting [25]. The intensivist must consider the patient’s comorbidities and past medical history because conditions such as inborn errors of metabolism are contraindications to the KD [25]. Patients should be assessed for metabolic pathologies such as mitochondrial enzyme deficiencies, acute pancreatitis, liver failure, pregnancy, intolerance to enteral nutrition, and recent infusions of propofol, which can cause a deadly infusion syndrome [25]. Notably, inconspicuous carbohydrate-containing drugs have hindered the achievement of ketosis in the case of new-onset refractory epilepticus [25]. Therefore, when using therapeutic ketosis, caution is advised regarding past, current, and future clinical states [25]. 

Referencing recent narrative reviews on the progress of KD therapy, we can further expand on its obstacles in clinical practice [11]. Of note, during early SE, patients are typically under intravenous anesthesia, drugs, and supplements, which may greatly impact the ketogenic ratio and therapeutic efficacy [11]. As such, there is a need to establish suitable, uniform ketogenic diet formulations and protocols that can be tailored to each patient. Another aforementioned obstacle includes the presence of carbohydrates from other sources, preventing the attainment of nutritional ketosis [11]. For example, medications like barbiturates and benzodiazepines contain large amounts of propylene glycol, and antibiotic agents such as vancomycin and trimethoprim-sulfamethoxazole necessitate the addition of 5% dextrose [11]. Furthermore, medications like glucocorticoids may prevent the generation of ketone bodies [11]. Lastly, the effects of KD on each primary illness need to be accounted for [11]. Nonetheless, cautious monitoring is necessary as KD may affect digestion, metabolism, and the urinary system [11]. Overall, the enteral KD is feasible and safe, but route- and illness-specific compositions must be further studied.

Therapeutic Ketosis in Sepsis and Infection

As previously discussed, many critical illnesses are associated with bioenergetic failure. Impaired lipid metabolism seems to play a significant role in sepsis, a common critical care diagnosis. The ‘obesity paradox’ is seen with obese patients having increased survival rates in intensive care units [23]. Therefore, can a diet high in fat (KD) improve outcomes? Due to associations between sepsis outcomes and lipids, ketone bodies are hypothesized to be beneficial in this critical illness [23]. In animal studies, septic mice with obesity demonstrated increased lipolysis and free fatty acid metabolism in the liver, in contrast to septic mice of normal weight [23]. Increased fatty acid metabolism and oxidation protected the obese mice against sepsis-induced muscle wasting and weakness [23]. On the contrary, when adipose triglyceride lipase (ATGL) was inhibited and lipolysis was decreased, the obese, septic mice experienced substantial muscle wasting and weakness [23]. Interestingly, administering high lipid doses or ketone bodies to septic mice of normal weight prevented sepsis-induced muscle weakness [23].

In an animal study recently published in Scientific Reports, a mouse model of sepsis-induced critical illness was used to test the safety and efficacy of ketone ester infusion for protection against muscle weakness [28]. The use of 3-hydroxybutyrate-sodium-salt causes toxicity from an excessive sodium load; therefore, the study’s objective was to investigate the ketone ester 3-hydroxybutyl-3-hydroxybutanoate (3HHB) as a well-tolerated and safe alternative [28]. The results showed that more bolus doses of 3HHB enantiomers significantly improved muscle force between the levels of 20-40 mmol/kg/day [28]. However, repetitive bolus doses caused higher mortality and severity of illness; therefore, the continuous infusion was used as a comparison and presented similar benefits with no increased risk of mortality or exacerbation of illness [28]. Notably, the positive effects were seen in the absence of toxicity caused by excessive sodium [28]. Overall, the animal study presented ketone ester continuous infusions as a potentially promising therapy for minimizing weakness in sepsis and critical illness [28]. Studies in humans are needed to validate these findings for clinical use.

The physiology behind KD benefits in sepsis is multifactorial. The administration of ketone bodies causes a decrease in both peripheral inflammation and neuroinflammation. This is due to ketone bodies’ suppression of oxidative stress and nonobese diabetic (NOD)-, leucine-rich repeat (LRR)-, and pyrin domain-containing protein 3 (NLRP3) inflammasome activity [23]. When given prophylactically, ketone bodies also affect the gut microbiome, decreasing Th17 intestinal pro-inflammatory cells, yet another possible beneficial effect in sepsis [23]. Another hypothesis is the involvement of peroxisome proliferator-activated receptor alpha (PPARα) [23]. In cecal ligation and puncture-induced sepsis, PPARα dysfunction is evident [22]. PPARα is an essential transcription factor that modulates gene transcription required for β-oxidation [22]. In sepsis, hepatic PPAR-α expression is suppressed, decreasing lipid oxidation [22]. This inability to oxidize lipids is a defect in the adaptive starvation response, leading to poor patient outcomes [22]. Many complications of sepsis are attributed to this abnormal metabolic adaptation, and low PPAR-α levels also correlate to the malnourishment seen in septic patients [22]. As PPARα plays a significant role in modulating ketogenesis, the potential use of ketone bodies as a therapeutic option has gathered interest [23]. Ketone bodies prevent damage from reactive oxygen species and provide an energy source to be utilized by extrahepatic tissues in septic mice [22]. In summary, ketone bodies may be protective against inflammation, muscle catabolism, and energy failure in sepsis. Similar metabolic disruptions have been detected in human sepsis [23].

In addition to sepsis due to various causes, the role of BHB in severe cases of respiratory viral infection was investigated, using influenza and SARS coronaviruses as examples [13]. Nutritional ketosis conveys potential benefits due to the pleiotropic role of ketone bodies at the interface of metabolism, inflammation, and aging, decreasing the burden of respiratory viral infection, especially in patients at risk of severe disease [13]. 

First, BHB can minimize oxidative stress, subsequently decreasing damage to the lung epithelium and the development of acute respiratory distress syndrome (ARDS) [13]. This is partially attributed to an increase in the cytoplasmic NADPH (nicotinamide adenine dinucleotide phosphate) supply [13]. In non-immune cell cytoplasm, NADPH has beneficial antioxidant activity through the reduction of oxidized redox couples [13]. Second, BHB stimulates the transcription factor Nrf2 to result in the expression of the antioxidant response element (ARE) gene [13]. This hinders histone deacetylase 1 (HDAC1) and HDAC2 function and, in turn, promotes the acetylation of histones locally at oxidative stress resistance genes (Foxo3a and Mt2) [13]. In numerous ARDS models, this stimulation of Nrf2 has proven to be protective [13]. As observed in two independent studies using chemically generated oxidative stress in a mouse model’s kidney, these two responses result in cytoprotection through the enhanced expression of protective genes [13]. Third, BHB and AcAc can work directly as antioxidants in vivo and in vitro models, scavenging a variety of free radicals [13]. Additionally, as discussed previously, BHB directly suppresses proinflammatory NLRP3 inflammasome activation, which is essential in the respiratory viral infection’s immune response [13]. Similarly, systemic inflammation is reduced due to BHB binding the HCAR2 receptor, hindering nuclear factor κB (NF-κB) signaling, and reducing NLRP3 inflammasome activity [13]. 

Ketones also help mitigate the complications of viral infections in multiple vital organs [13]. Influenza infection poses an acute and chronic risk of cardiac damage and associated deaths; ketone bodies may help minimize these effects [13]. Evidence from mouse models and adults with heart failure conveys that cardiomyocytes undergo adaptation to preferentially utilize ketone bodies [13]. In fact, the absence of the enzymes needed to metabolize BHB causes pathological cardiac remodeling [13]. According to ex vivo studies, ketone bodies cause an approximate 24% increase in cardiac efficiency in comparison to solely metabolizing glucose [13]. According to in vivo and human clinical studies, intravenous infusion of ketone bodies causes an enhancement in cardiac function with elevated ejection fractions [13]. Respiratory-related protection is also beneficial, as COVID-19, influenza, and ARDS typically lead to hypoxemia [13]. If unresponsive to treatment, the hypoxemia commonly progresses to multiorgan failure and mortality [13]. Increased ketone levels minimize hypoxia-related tissue damage, especially in the brain [13]. In models with ischemic and hypoxic brain insults, exogenous BHB sustained adequate cerebral ATP levels, enhanced the survival of neurons, lessened the volume of infarcts, reduced edema, and led to higher performance and cognition [13]. Additionally, KD can also affect the gut microbiota in both mice and humans, and this is another hypothesized mechanism involved in enhancing immunity to viral respiratory infections [13]. 

As discussed previously, muscle function can be preserved through KD administration; this is especially important in respiratory illness due to multiple reasons. Life after critical illness and ARDS is often accompanied by decreased muscle mass, disability, and impaired function; these are significant predictors of morbidity and mortality [13]. Weakness is seen in half of patients after recovery; specifically, influenza leads to muscle catabolism in a dose-dependent mechanism with increased proinflammatory cytokines (IL-6 and TNF-α) and myostatin and suppressed anabolic signaling through low insulin growth factor 1 levels (IGF-1) [13]. A similar response is seen in COVID-19 with resultant myositis, myalgia, and muscle breakdown [13]. Another form of dangerous muscle atrophy is the ubiquitin-associated diaphragmatic atrophy seen in patients on ventilation, which contributes to morbidity and mortality up to one year after recovery [13]. The anti-catabolic role of exogenous ketones is useful in inflammatory settings of various etiologies and ubiquitin-driven atrophy [13]. Evidence shows that one dose of ketones caused a 46% reduction in infection-induced tissue catabolism and quickened recovery [13]. This is thought to be due to BHB’s interaction with NF-κB and HCAR2, as aforementioned [13].

Another consequence of severe viral infection in intensive care unit admissions is delirium [13]. It is an acute confusional state seen in most critically ill patients [13]. Delirium independently increases mortality, length of intensive care unit stays, and long-term decline of cognitive function [13]. Some of the previously discussed mechanisms such as modulation of NF-κB and NLRP3 can reduce systemic and cerebral inflammation [13]. Preventing hypoxia and avoiding hypoglycemia may further preserve neuronal health [13]. Drawing from the KD’s role in molecular mechanisms, the administration of KD especially in older patients and patients with decreased cognitive function, may perhaps provide a protective effect against delirium and enhance resolution [13].

Therapeutic Ketosis and Glycemic Control

It is imperative to prevent both hypoglycemia and severe hyperglycemia to increase favorable outcomes in patients in the intensive care unit. Evidence suggests administration of ketone bodies may lessen glycemic dysregulation and its associated complications in high-risk patients [13]. In various animal studies of multifactorial inflammatory disease, exogenous ketones in acute and chronic settings decreased glucose levels, minimized injury attributed to hypoglycemia, and yielded more favorable illness outcomes [13]. 

As discussed previously, nutritional ketosis results in blood glucose stabilization and decreases the need for insulin [29]. This is increasingly observed in patients with certain characteristics [12]. A recent review found that formulas with low-carbohydrate and high-fat (LCHF) ratios augmented glycemic control, especially in diabetic or hyperglycemic patients with critical illnesses [12]. The paper reviewed four randomized controlled trials that explored the difference in effects between standard formulas and LCHF formulas on blood sugar levels in critical illness [12]. Two of the studies found that LCHF formulas caused a significant enhancement in glycemic control [12]. However, possibly due to variations in patient inclusion criteria in specifying hyperglycemia and diabetes, two studies did not find significant results [12]. Subgroup analyses were conducted, and the positive effects of LCHF formulas seen in diabetic patients were in line with previous guidelines set by Critical Care Nutrition and ASPEN [12]. Further studies with specified formulation ratios, ketosis measurements, and standardized inclusion criteria are warranted. 

Adverse Effects of the Ketogenic Diet

The absolute contraindications to KD in adults are few. Due to the necessity of fat metabolism, disorders of fatty acid transport and oxidation, liver failure, porphyrias, organic acidurias, and certain inborn errors of metabolism are contraindications [21]. Adverse effects are mostly mild and include weight loss, gastrointestinal effects such as constipation, changes in lipid profiles, and menstrual alterations [21]. Vitamin and trace elements such as selenium, zinc, and carnitine deficiencies can also be seen [21]. Of note, more studies are warranted to explore the adverse effects of KD in various disease states.

Limitations

This systematic review was limited to reports from four databases published between 2019 and 2024 and articles in the English language. Moreover, due to the retrieval of only free full-text papers, relevant data may have been precluded. The majority of reports covered in this systematic review were narrative reviews; although evidence of therapeutic benefit is promising, multicenter, randomized, controlled studies with large sample sizes are necessary to validate the use of KD as a treatment. As this review focused on critically ill subjects, limited evidence of a novel therapeutic approach for high-risk patients is expected. Moreover, there is a lack of human clinical trials and studies focused on the adult age group. As guidelines differ and the therapeutic approach is novel, different types of KD vary between studies, with unstandardized formulation proportions (carbohydrates, fat, and protein). Future research should include randomized controlled trials with large sample sizes and adult patients; standardized KD formulations, detailed routes, and specific blood level confirmation of ketosis should be included.

## Conclusions

In this systematic review, the use of nutritional ketosis as a potential therapeutic approach for patients with critical illnesses was explored. With critical illness posing multifaceted challenges and ketosis having a multitude of pleiotropic effects, this may be a promising innovation. Critical illness commonly implicates states of bioenergetic failure, inflammation, and oxidative stress; ketosis has the potential to effectively and safely mitigate these pathological states. In status epilepticus, ketosis may aid in the cessation of seizures effectively and with reduced adverse effects. In sepsis and viral infection, ketosis may provide anti-inflammatory and antioxidant benefits, protect muscle, preserve cardiac and cerebral function, reduce hypoxic damage, prevent delirium, and improve infection outcomes. Regarding glycemic control, KD may improve glucose regulation in various patient subgroups. Overall, current evidence presents a promising reduction in morbidity and mortality, supporting nutritional ketosis as a potential therapeutic approach in critically ill patients. Today, the utility of therapeutic ketosis can be postulated by its impact on molecular mechanisms and outcomes in smaller studies. Some suggestions for future research include studying the effect of KD with a focus on specific diagnoses; for example, larger human trials investigating KD use in sepsis, heart failure, and brain injury can provide further impactful evidence. The investigation of nutritional ketosis as a therapy is captivating, as it entails utilizing a variant of the body's innate metabolic pathways to treat illness. In the end, research in this area may provide significant innovation and benefit to a large yet vulnerable population in a feasible and accessible way.
